# Audiovisual n-Back Training Alters the Neural Processes of Working Memory and Audiovisual Integration: Evidence of Changes in ERPs

**DOI:** 10.3390/brainsci13070992

**Published:** 2023-06-24

**Authors:** Ao Guo, Weiping Yang, Xiangfu Yang, Jinfei Lin, Zimo Li, Yanna Ren, Jiajia Yang, Jinglong Wu

**Affiliations:** 1 Department of Psychology, Faculty of Education, Hubei University, Wuhan 430062, China; psyyxf@163.com (X.Y.); loye_lin@163.com (J.L.); 2 Cognitive Neuroscience Laboratory, Graduate School of Interdisciplinary Science and Engineering in Health Systems, Okayama University, Okayama 700-8530, Japan; godcouldcry@163.com (A.G.); pprr2x1y@s.okayama-u.ac.jp (Z.L.); yang@okayama-u.ac.jp (J.Y.); 3 Brain and Cognition Research Center (BCRC), Faculty of Education, Hubei University, Wuhan 430062, China; 4 Department of Psychology, College of Humanities and Management, Guizhou University of Traditional Chinese Medicine, Guiyang 550003, China; yanna052267213@163.com; 5 Applied Brain Science Lab., Graduate School of Interdisciplinary Science and Engineering in Health Systems, Okayama University, Okayama 700-8530, Japan; 6 School of Medical Technology, Beijing Institute of Technology, Beijing 100811, China

**Keywords:** audiovisual n-back, training, audiovisual integration, ERPs, training effect, transfer effect

## Abstract

(1) Background: This study investigates whether audiovisual n-back training leads to training effects on working memory and transfer effects on perceptual processing. (2) Methods: Before and after training, the participants were tested using the audiovisual n-back task (1-, 2-, or 3-back), to detect training effects, and the audiovisual discrimination task, to detect transfer effects. (3) Results: For the training effect, the behavioral results show that training leads to greater accuracy and faster response times. Stronger training gains in accuracy and response time using 3- and 2-back tasks, compared to 1-back, were observed in the training group. Event-related potentials (ERPs) data revealed an enhancement of P300 in the frontal and central regions across all working memory levels after training. Training also led to the enhancement of N200 in the central region in the 3-back condition. For the transfer effect, greater audiovisual integration in the frontal and central regions during the post-test rather than pre-test was observed at an early stage (80–120 ms) in the training group. (4) Conclusion: Our findings provide evidence that audiovisual n-back training enhances neural processes underlying a working memory and demonstrate a positive influence of higher cognitive functions on lower cognitive functions.

## 1. Introduction

Working memory is a cognitive system used for the temporary maintenance and manipulation of information [1]. Modality-specific working memory training using functional magnetic resonance imaging (fMRI) induces changes in neural activation and improves working memory performance. Some fMRI reports have shown that visual working memory training leads to enhanced prefrontal and parietal activations responsible for visual working memory storage, which is associated with the training effect [2]. Training-induced activation changes have also been observed in auditory transfer tasks following auditory working memory training, where the right inferior frontal regions related to the maintenance of auditory information are engaged in the improvement of working memory [3]. The differences between modality-specific working memory training have also been explored. Following two weeks of separate visual and auditory n-back training tests, researchers examined the transfer effects of these two types of working memory training on an untrained visual working memory task based on fMRI. Only visual n-back training induced additional activation in the right middle frontal regions during the untrained visual task; such a decreased activation was not observed after auditory n-back training. The authors further confirmed that the right middle frontal regions are specific to the maintenance and manipulation of visual information [4]. These studies suggest that modality-specific training produces specific activation changes in the working memory network. However, according to the multiple-component model, working memory consists of a central executive and two components specialized for maintaining modality-specific information [1]. The phonological loop is specialized for retaining auditory and phonological information, while the visuospatial sketchpad is used to retain visual–spatial and nonspatial information. Working memory relies on the processing efficiency of the visuospatial sketchpad and phonological loop [5]; thus, facilitating the storage and processing of these components may enhance working memory performance [3,4].

Dual n-back training is in line with a multiple-component model, requiring the simultaneous processing of visual and auditory stimuli. Visual stimuli consist of blue squares in eight different locations and presented one by one. Auditory stimuli consist of the sounds of single letters. Working memory contents are monitored and updated in these two modalities separately. There has been considerable interest in the dual n-back task, given its potential to improve working memory, and the transfer effects on fluid intelligence, executive function, and attention [6]. However, conflicting results regarding dual n-back training have been reported. That is, some researchers have found that this task may not promote a training effect because employing incongruent visual and auditory information leads to competition between modalities and interferes with the participant’s response [7]. For example, in some dual n-back training tasks, greater training and transfer effects were not observed [8,9]. In more detail, training on a single n-back task showed a transfer effect on fluid intelligence similar to that of dual n-back training.

Notably, visual and auditory information may be related, leading to an audiovisual facilitation effect [10]. This effect demonstrates that the merging of the related visual and auditory stimuli is integrated into a coherent percept and leads to enhanced information processing at both the behavioral and neural levels [11,12]. That is, when participants memorized audiovisual, visual, and auditory stimuli, respectively, the accuracy and response time of working memory performances were better overall during the audiovisual stimuli presentation relative to the unimodal stimuli presentation in working memory [5]. Some researchers using ERPs found that the latency of the P3 component evoked during the audiovisual stimuli presentation occurred earlier than the latency evoked during the unimodal stimuli presentation in working memory. The earlier latency indicated faster cognitive processing times during the audiovisual stimuli presentation in working memory [13]. The cognitive load theory argues that the capacity to handle information increases by using both visuospatial sketchpad and phonological-loop components [14]. When all the information has to be processed by the visuospatial sketchpad, its capacity is easily overloaded. The simultaneous presentation of related auditory information may reduce some of the load on the visuospatial sketchpad by shifting it to the phonological loop, thereby enhancing working memory performance [14]. Considering the audiovisual advantage in working memory processes, some researchers have examined the behavioral impact of working memory training with audiovisual stimuli on working memory performance [15]. Two groups of participants completed audiovisual and visual n-back training tasks. Their behavioral results indicate that the group with audiovisual n-back training not only exhibits equal training gains, but also potentially exhibits transfer effects on a complex working memory span task compared to the unimodal n-back training group. However, this research did not address the neural effect of working memory training with audiovisual stimuli [15]. Providing neural evidence may better reveal the potential reasons for why the audiovisual n-back training is effective.

With regard to the neural evidence, training effects on certain brain regions have been determined in previous studies, including activation changes in the frontal and partial regions and connectivity between the prefrontal–parietal network [2,16]. Although it is critical to identify specific regions that are influenced by working memory training, fMRI may not fully reveal the subcomponents of working memory processing affected by training. Recent studies with ERPs have demonstrated that visual and dual n-back working memory training tasks modulate separate components of the working memory process [17,18]. They found greater N2 amplitudes, which is related to mismatch/match identification [19]. The subsequent P3 amplitude, which indexes working memory updating, was also enhanced after training [20]. This raises the question of whether working memory training with audiovisual n-back could influence these ERP components (N200, P300) and enhance working memory performance. Previous studies have explored the associated brain activity between different processing stages in a delayed-matching working memory task [21]. They found that similar activations between early encoding and later maintenance in the lateral prefrontal and visual cortexes was related to an improved working memory performance. The results indicate that similar brain activity during different processing stages of the working memory underlies the improvement to working memory [21]. In this study, early audiovisual processing at the encoding stage and subsequent mismatch identification and updating were involved in the audiovisual n-back training task. Audiovisual processing produces similar brain activation in the frontal region as mismatch identification and similar brain activation in the central region as updating [19,22,23]. This suggests that working memory with audiovisual stimuli may induce a successful working memory performance. According to the information degradation hypothesis, sensory and higher-order cognitive processing tasks share limited cognitive resources [24]. Under this hypothesis, changes in one of the systems can influence the efficiency of the other, that is, dedicating too many cognitive resources to perceptual processing may result in insufficient resources for subsequent higher cognitive processing, such as mismatch identification and working memory updates. The presentation of audiovisual stimuli at the perceptual stage may provide more resources for subsequent working memory processing and enhance processing efficiency. Therefore, we hypothesize that audiovisual working memory training may induce enhanced N2 and P3 components, thereby enhancing working memory performance.

Despite the evidence that working memory training improves a variety of cognitive functions, the training-induced transfer effect is still debated. Recent studies have suggested that the transfer to untrained working memory tasks may be consistently observed; however, the transfer effect on fluid intelligence is not preserved [15,25,26,27]. This conflict may result from the ambiguity over whether there is an overlap in the brain regions involved in training and transfer tasks. In particular, some fMRI studies have found that working memory updating training yields transfer to an n-back task, in which the updating process is engaged, but no transfer to a Stroop task, which involves the inhibition process [28]. Further investigations of neural activation showed that the overlap of striatal activation between training and n-back tasks determined the transfer, indicating that a transfer effect is expected if the training and transfer tasks have specific overlapping brain regions [28]. Some neural evidence has further demonstrated that parts of working memory circuity, such as the left intraparietal region, have been linked to audiovisual processing [29], indicating that the transfer effect of working memory training on audiovisual processing is possible. Moreover, the stronger the correlation between the working memory and this alternative cognitive ability, the greater the expected transfer effect [30]. Audiovisual processing research has shown that there is a connection between sensory perceptions and cognitive abilities, reflected by older adults with greater working memory capacities exhibiting a better performance in audiovisual processing [31,32]. Therefore, we hypothesize that training that successfully improves the working memory also directly affects audiovisual processing.

To investigate the training and transfer effects, we designed an audiovisual n-back working memory task that includes related visual and auditory information. The audiovisual stimuli we use in the training task combine animal images and sounds, where the long-term memory might be involved. Prior to (pre-test) and following training (post-test), P300 and N200 components were elicited by an audiovisual n-back task with 3 levels (1-, 2-, or 3-back) in both groups. By comparing the P300 and N200 components between the pre- and post-tests, we could determine whether working memory performance could be improved. By testing the transfer effects, the current study verifies whether working memory training facilitates audiovisual processing using an audiovisual discrimination task. In summary, the purpose of our study is to investigate whether audiovisual working memory training can facilitate working memory and audiovisual processing, including both behavioral performances and neural outcomes. Our study reveals the neural mechanisms of audiovisual working memory training, presenting important implications for the development of effective cognitive training programs.

## 2. Methods

### 2.1. Participants

Thirty-seven healthy young adults from Hubei University were recruited for the current study. They were randomly assigned to a passive control group (11 males and 6 females, mean age = 20.65 years old, SD = 1.7) and an audiovisual n-back training group (9 males and 11 females, mean age = 20.52 years old, SD = 1.9). All the participants reported having normal or corrected-to-normal vision and hearing abilities. The two groups were comparable in terms of education and fluid intelligence (Raven’s Advanced Progressive Matrices).

### 2.2. General Procedure

**Figure 1:**
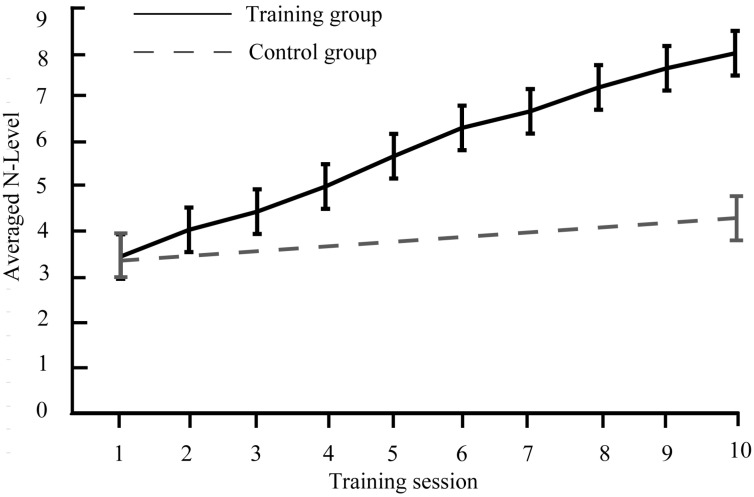
The performance of audiovisual n-back training across every training session. For each session, the averaged n-back level is presented.

**Figure 2:**
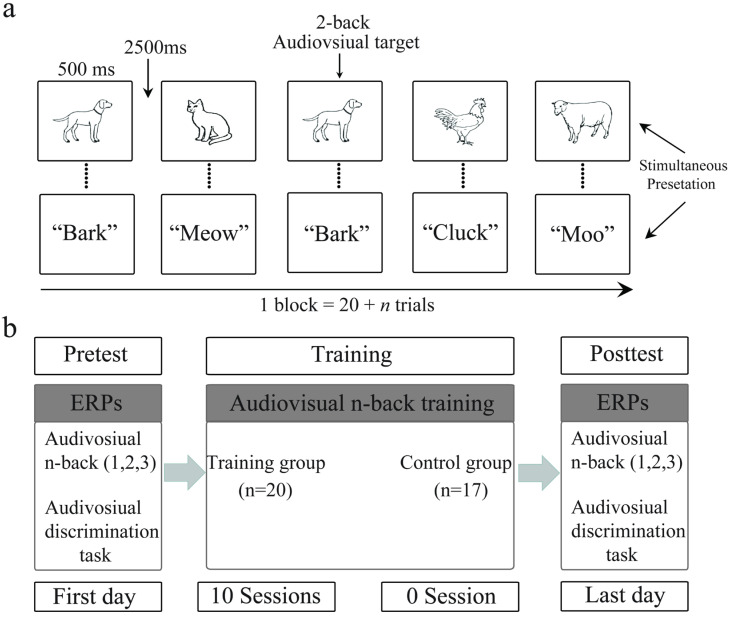
(**a**) Example of a 2-back task in the audiovisual working memory training test. Each stimulus was presented for 500 ms and presented both auditory and visual information (i.e., participants can both hear and see the animals). Participants were instructed to determine whether the presented animal matched the animal presented in the two previous trials. (**b**) Schematic description of the study design. Both groups performed the audiovisual n-back (1-, 2-, and 3-back) and audiovisual discrimination tasks pre- and post-test (ERPs). During training, the training group participated in an adaptive audiovisual n-back task, whereas the control group did not receive any training.

The training group participated in 10 training sessions containing audiovisual n-back tasks over 2 weeks (5 training sessions per week), whereas the control group underwent no training during this time. For the specific processing of 10 training sessions, see Figure 1. Each training session was approximately 50 min, with about 8 h of total training. During the pre- and post-tests, the participants were required to perform audiovisual n-back (1-, 2-, or 3-back) and discrimination tasks, and the ERPs data were collected. The audiovisual n-back task (1-, 2-, or 3-back) was adopted to assess the training effect and the discrimination task was used to assess the transfer effect. The general procedure can be found in Figure 2b. After comparing the behavioral and neural differences between the training and control groups during the pre- and post-tests, the training and transfer effects induced by audiovisual n-back training were determined. 

#### 2.2.1. Training Task

The adaptive audiovisual n-back task was designed to promote learning (see Figure 2a). This task consisted of simultaneously presenting visual and auditory stimuli. The visual stimuli were black-and-white-line images of animals chosen by Snodgrass and Vanderwart [33], and the auditory stimuli were the corresponding animal sounds selected from a website (http://www.findsounds.com, 10 June 2021). The visual and auditory stimuli were presented simultaneously for 500 ms, followed by an interstimulus interval of 2500 ms. Each training session began with the 1-back condition. The task was based on the adaptive principle: if participants provided correct responses for at least 90% of the trials, the task advanced to the next level (e.g., from 1- to 2-back). On the other hand, if participants provided correct responses to ≤80% of trials, the task difficulty was reduced (e.g., from 2- to 1-back). Participants responded by pressing the left mouse button when the current stimulus matched the one presented n steps back, or the right mouse button when the current stimulus did not match the one presented n steps back. Each training session contained 20 blocks. Each block consisted of 20 + n trials with 6 targets (matched trials) and 14 nontargets (unmatched trials). After training in each block, participants received feedback on their performance.

#### 2.2.2. Training and Transfer Outcomes

For the pre- and post-tests, the participants were instructed to perform a modified version of the audiovisual n-back task. This task provided no feedback, presented the same stimuli as the training task, was not adaptive (only three n-back conditions: 1-, 2-, and 3-back), and was adopted to measure the training effectiveness using EEG and behavioral data. This task consisted of 15 blocks (5 blocks per level).

The subjects also performed an audiovisual discrimination task, including target and standard stimuli, for both pre- and post-tests. The source of the stimuli was the same as that in the training task. In the experiment, the stimulus types consisted of target and standard stimuli. The target stimuli included a visual (the image of a dog), auditory (the sound of a dog), and audiovisual targets (the presentation of the visual and auditory targets simultaneously). The standard stimuli included visual (the image of a cat), auditory (the sound of a cat), and audiovisual standard stimuli (the presentation of the visual and auditory targets simultaneously). During the experiment, 3 block tests were conducted. Each block contained 36 target stimuli (12 auditory, 12 visual, and 12 audiovisual) and 144 standard stimuli (48 auditory, 48 visual, and 48 audiovisual). The stimuli were randomly presented for 500 ms with an interstimulus interval of 1000 ms. Participants were instructed to fix their gaze on the center of the computer screen, where the stimuli were presented. They were asked to press the left mouse button as quickly and accurately as possible when target stimuli were presented.

### 2.3. EEG Data Recording and Preprocessing

EEG activity was recorded with an EEG system (BrainAmp MR plus, Gilching, Germany) using a 32-electrode EEG cap (Easy-cap, Herrsching Breitbrunn, Germany). The reference electrode was positioned at FCz. Horizontal electrooculogram (EOG) data were monitored with an electrode positioned at the outer canthi of the left eye, and the vertical EOG was monitored with an electrode positioned roughly 1 cm below the right eye. All the signals were digitized with a sampling rate of 1000 Hz. During EEG recording, the impedances of all electrodes were kept below 5 kΩ.

The offline analysis for EEG data was conducted using functions in EEGLAB and ERPLAB under MATLAB software (2016a). The position of EEG electrodes was based on the 32-channel montage of the international 10–20 system. All data were re-referenced to the mastoid electrodes (TP9, TP10) and bandpass filtered from 0.1 to 30 Hz at a downsampling rate of 500 Hz. The ERPs elicited by matched trials were divided into epochs starting from 200 ms pre-stimulus to 1000 ms post-stimulus (600 points), with baseline corrections performed from 200 ms pre-stimulus. Each trial was corrected using its own baseline. The subsequent averaging stage rejected epochs with large artifacts if the voltage exceeded ±100 µV. Then, all the remaining trials were averaged separately for different n-back conditions (1-, 2-, and 3-back), and the ground-averaged data were also acquired by averaging each electrode under each n-back condition across all participants. For N200 and P300 components, peak detection was performed on averaged waveforms of each participant. Based on the previous studies, the amplitude of the N200 component was regarded as the peak that occurred at around 200–350 ms, whereas the amplitude of the P300 component was quantified as the peak that occurred at approximately 300–600 ms [18,34]. These ERP components’ amplitudes were averaged across the electrodes in each brain region, which may have reduced the noise resulting from the instability of individual electrodes [18].

The EEG recording procedure for the transfer task was identical for the audiovisual n-back task. The ERPs elicited by standard trials were divided into epochs starting from 100 ms pre-stimulus and 800 ms post-stimulus (450 points), with baseline corrections performed from 100 ms pre-stimulus. When the voltage exceeded ± 100 µV, epochs were considered to be contaminated by large artifacts. Then, these epochs were rejected. The remaining epochs were averaged for each stimulus condition (auditory, visual, and audiovisual), and ground-averaged data were also acquired by averaging each electrode under each stimulus condition across all participants. 

### 2.4. Data Analysis

We analyzed the effect of training by examining the behavioral differences (accuracy and reaction time) between pre- and post-test subjects. Repeated-measures ANOVAs with session (pre- and post-tests) × load (1-, 2-, 3-back) × group (training and control groups) were performed separately for reaction time (RT) and accuracy in the training task. To assess the accuracy, the sensitivity index (*d’*) was calculated for every participant and averaged for the of pre- and post-test stages. *d’* was estimated as the difference between the hit and false-alarm rates. The reaction time was calculated as the average of the sum of reaction times for correct responses to match trials and correct responses to unmatched trials. To compare the training gains among different working memory loads, we also conducted repeated-measures ANOVAs with loads (1-, 2-, 3-back) as the within-subject factor and group (training and control groups) as the between-subject factor. The dependent variables were the difference in the reaction time and accuracy between the post- and the pre-tests. For the transfer effect, repeated-measures ANOVAs with session (pre- and post-tests) × modality (V, A, AV) × group (training and control groups) were conducted for accuracy and reaction time. Accuracy was the proportion of correct responses to target stimuli relative to total target stimuli. Reaction time was based on the correct responses to target stimuli. 

To examine neural changes after training, we selected the following regions of interest (ROIs) based on the previous studies [35]: frontal (F3, Fz, F4), central (C3, Cz, C4), and parietal (P3, Pz, P4). The amplitudes of the ERPs components (N2, P3) were averaged across electrodes within each ROI, which may reduce the noise resulting from the instability of individual electrodes [18]. For each ERP component, repeated-measures ANOVAs were conducted separately to examine the amplitudes in each region. The content of the analysis included (pre- and post-tests) × group (training and control groups) × load (1-, 2-, 3-back). 

To obtain the transfer outcomes, the following equation was used to quantify the transfer effect: ERP(AV) − [ERP(A) + ERP(V)]. This equation subtracted the summed ERPs for unimodal visual and auditory trials from the ERPs for audiovisual trials at each time bin for each electrode [12]. Then, the amplitudes of the difference [AV − (A + V)] were compared with 0 at each time point from 0 to 500 ms, using one-sample *t*-tests. If more than 12 consecutive time points were significantly different from zero (α < 0.05), audiovisual integration was considered to have occurred. This criterion ensures the reliability of results when a large number of *t*-tests are conducted [36]. Based on the results of the t-test, three integration time intervals (80–120, 170–210, and 350–390 ms) and three ROIs (frontal: Fz, F3, F4; central: C3, C4, Cz; and parietal: P3, P4, Pz) were selected for further analysis. In addition, the amplitude of ERPs was averaged across electrodes within each ROI. Repeated-measures ANOVAs with sessions (pre- and post-tests) × ROIs (frontal: Fz, F3, F4; central: C3, C4, Cz; and parietal: P3, P4, Pz) × group (training and control groups) were conducted. The dependent variable was the amplitude difference of [AV − (A + V)].

## 3. Results

### 3.1. Behavioral Results

#### 3.1.1. Training Outcomes

**Figure 3:**
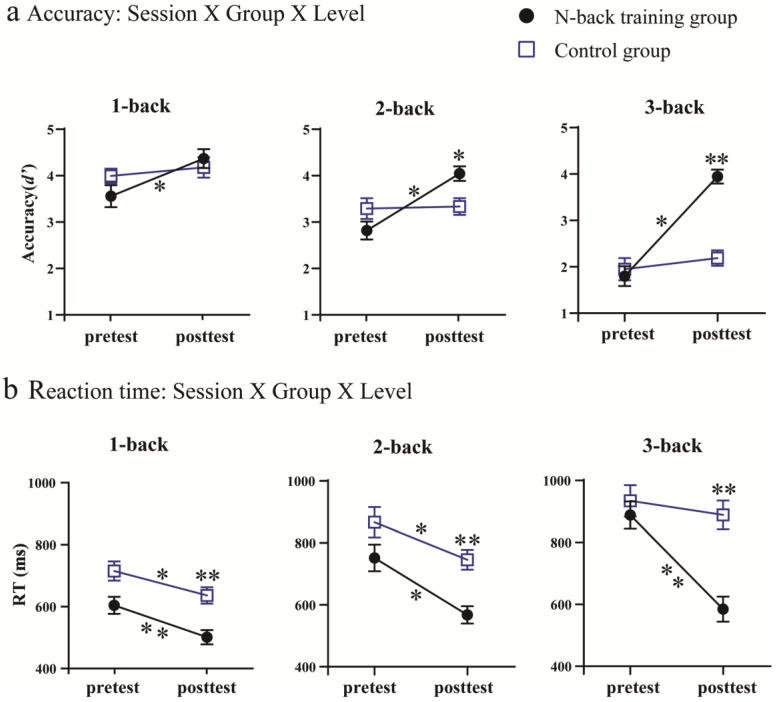
Changes in performances between pre- and post-tests. (**a**) Accuracy is depicted for 1-, 2-, and 3-back sessions. (**b**) Reaction times are shown for 1-, 2-, and 3-back sessions. The black circles represent the training group and the blue squares represent the control group. Error bars demonstrate the standard error of the mean (SEM). The asterisks along a trendline indicate the difference between the pre- and post-tests, and the asterisks above the black circles or blue squares indicate the differences between the different groups pre- or posttest, * *p* < 0.05, ** *p* < 0.01.

The ANOVAs based on accuracy revealed a significant main effect of session [*F*(1, 35) = 58.299, *p* < 0.001, η_p_^2^ = 0.625], suggesting that the post-test accuracy (*d’*) increased compared to the pre-test accuracy. The significant main effect of the load was also observed [*F*(2, 70) = 80.937, *p* < 0.001, η_p_^2^ = 0.698], indicating that the accuracy decreased with an increased working memory load (3-back < 2-back < 1-back). Moreover, a two-way interaction between the session and load was observed [*F*(2, 70) = 7.019, *p* = 0.002, η_p_^2^ = 0.167]. A post hoc analysis using pairwise comparisons with a Bonferroni correction indicated that participants were more accurate post-test than pre-test across all working memory loads, *ps* < 0.010. Additionally, a significant interaction between the load and group was observed [*F*(2, 70) = 7.679, *p* = 0.001, η_p_^2^ = 0.180]. After the post hoc analysis using pairwise comparisons with a Bonferroni correction, we found that the accuracy decreased significantly with an increased load in both groups, *ps* < 0.010. Importantly, there was a significant interaction between session and group [*F*(1, 35) = 37.152, *p* < 0.001, η_p_^2^ = 0.515]. The post hoc analysis using pairwise comparisons with a Bonferroni correction showed that the accuracy was significantly improved in the training group after training, *p* < 0.001. This improvement was not observed in the control group, *p* = 0.314. There was a significant three-way interaction [*F*(2, 70) = 5.270, *p* = 0.007, η_p_^2^ = 0.131]. Post hoc analysis using pairwise comparisons with a Bonferroni correction showed that the training group exhibited a significant accuracy improvement post-test compared to pre-test across all working memory loads, *ps* < 0.001. However, when comparing the pre- and post-tests in the control group across all working memory loads, there was no significant difference (see Figure 3a), *ps* > 0.05. In addition, although no significant differences were observed between groups for all working memory loads during the pre-test, the training group had significantly better accuracy results than the control group in the 2- and 3-back conditions post-test (see Figure 3a), *ps* < 0.01.

The analyses of reaction times showed that there was a significant main effect of the session [*F*(1, 35) = 37.077, *p* < 0.001, η_p_^2^ = 0.514], with the post-test reaction time being faster than the pre-test. The main effect of the load was significant [*F*(2, 70) = 51.315, *p* < 0.001, η_p_^2^ = 0.595], with the reaction time increasing with a higher working memory load (1-back < 2-back < 3-back). The significant main effect on the group was also observed [*F*(1, 35) = 13.848, *p* = 0.001, η_p_^2^= 0.283], indicating that the reaction time of the training group was faster than that of the control group. Moreover, a significant interaction between session and load was observed [*F*(2, 70) = 5.373, *p* < 0.01, η_p_^2^ = 0.133]. A post hoc analysis using pairwise comparisons with a Bonferroni correction revealed that the post-test reaction time decreased significantly compared to the pre-test across all working memory loads, *ps* < 0.05. It was worth noting that an interaction between session and group was observed [*F*(1, 35) = 6.366, *p* < 0.05, η_p_^2^ = 0.154]. The post hoc analysis using pairwise comparisons with a Bonferroni correction showed that there was no significant difference between the training and control groups pre-test, *p* = 0.080. However, the reaction time significantly decreased in the training group post-test, *p* < 0.001. The load × group interaction was not significant [*F*(2, 70) = 0.800, *p* = 0.453, η_p_^2^ = 0.022]. Notably, the three-way interaction between session, load, and group was significant [*F*(2, 70) = 11.177, *p* < 0.001, η_p_^2^ = 0.242]. A post hoc analysis using pairwise comparisons with a Bonferroni correction demonstrated that the training group showed a significant reaction-time improvement across all working memory loads, *ps* < 0.001 (see Figure 3b). Similar results were observed in the 1- and 2-back conditions for the control group, *ps* < 0.01. Moreover, post-test, the training group showed significantly decreased reaction times for all working memory loads compared to the control group, *ps* < 0.05 (see Figure 3b). However, the two groups did not show a significant difference pre-test, *ps* > 0.05.

#### 3.1.2. Training Gain

**Figure 4:**
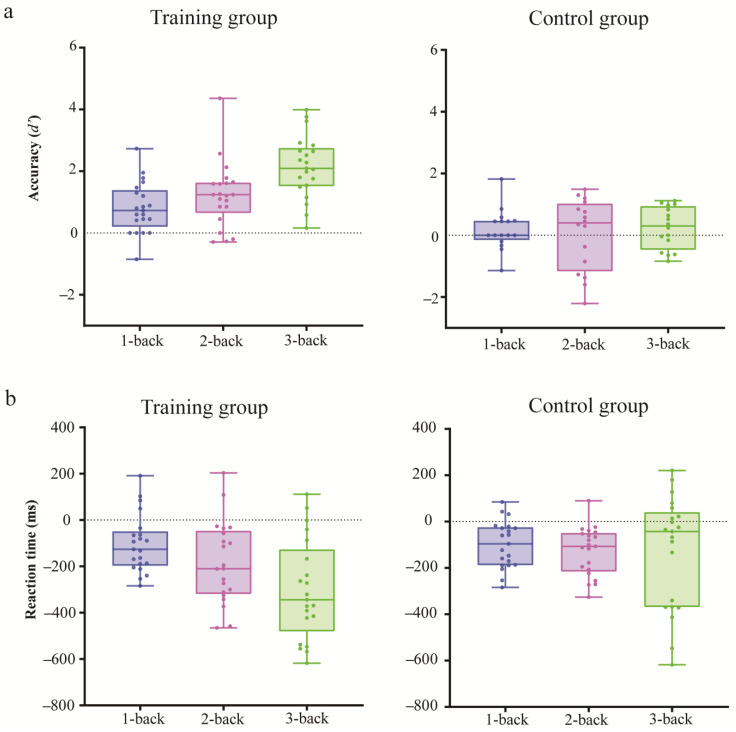
Box plots of training gains for accuracy (**a**) and reaction time (**b**) across 1-, 2-, and 3-back conditions in training and control groups. The solid line inside the box represents the median. Upper and lower quartiles are represented by the upper and lower borders, respectively.

The ANOVA for training gain in accuracy (*d’*) showed that the main effect of load was significant [*F*(2, 70) = 7.01, *p* < 0.01, η_p_^2^ = 0.17], indicating that the training gain in the 3-back condition was greater than that in the 2- and 1-back conditions. The main effect of the group was also significant [*F*(1, 35) = 37.20, *p* < 0.001, η_p_^2^ = 0.52], presenting greater training gains in the training group than in the control group. The interaction between load and group was significant [*F*(2, 70) = 5.28, *p* < 0.01, η_p_^2^ = 0.13]. A post hoc analysis using pairwise comparisons with a Bonferroni correction showed that the training gain of the training group in the 3-back condition was greater than that in the 2-back and 1- conditions, *ps* < 0.01. However, the training gain of the control group across the 1-, 2-, and 3-back conditions was not significantly different, *ps* > 0.050. In terms of the load, a greater training gain was achieved in the training group than in the control group across all working memory levels, *ps* < 0.05. The detailed training gain results for accuracy are shown in Figure 4a.

The ANOVA for training gain with reaction time demonstrated that the main effect of load was significant [*F*(2, 70) = 5.373, *p* = 0.07, η_p_^2^ = 0.133], suggesting that the training gains in the 2- and 3-back conditions were significantly better than that in the 1-back condition. The main effect of the group was also significant [*F*(1, 35) = 6.366, *p* = 0.016, η_p_^2^ = 0.154], with greater training gains in the training group than in the control group. The interaction between load and group was significant [*F*(2, 70) = 11.177, *p* < 0.001, η_p_^2^ = 0.242]. A further post hoc analysis using pairwise comparisons with a Bonferroni correction indicated that the training gain of the training group in the 3-back condition was greater than that in the 2- and 1-back conditions, *ps* < 0.050. Similar to the results of the training gain in accuracy, there was no difference in training gain results across the 1-, 2-, and 3-back conditions in the control group, *ps* > 0.050. In terms of the load, a greater training gain in the training group was observed only in the 3-back condition compared to the control group, *p* < 0.001. In the 1- and 2-back conditions, no significant differences between the two groups were observed, *ps* > 0.050. The detailed training gain results for the reaction time are shown in Figure 4b.

#### 3.1.3. Transfer Outcomes

The ANOVA for reaction time showed that the main effect of modality was significant [*F*(2, 70) = 135.602, *p* < 0.001, η_p_^2^ = 0.790], indicating that the reaction time to audiovisual stimuli was quicker than that to visual and auditory stimuli, see Table 1. No other interaction was observed (*ps* > 0.050).

Similar to the reaction-time results, only the significant main effect of modality was observed [*F*(2, 72) = 53.81, *p* = 0.00, η_p_^2^ = 0.60], indicating that the accuracy of responses to audiovisual stimuli was greater than that to visual and auditory stimuli, see Table 1. No other significant main or interaction effects were observed (*ps* > 0.050).

### 3.2. ERP Results

#### 3.2.1. Training Outcomes

**Figure 5:**
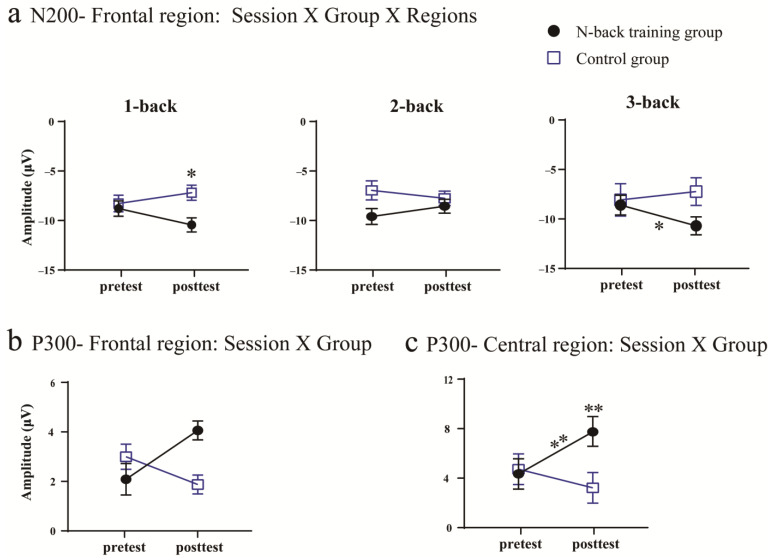
Training-related effects on ERP components. Changes in the N200 amplitude in the central region (**a**) and the P300 amplitude in the frontal region (**b**) and central region (**c**) are depicted. Error bars show the SEM. The asterisk along the trendline indicates the difference between the pre- and post-test results, and the asterisks above the black circles or blue squares indicate the differences between different groups pre- or post-test, * *p* < 0.05, ** *p* < 0.01.

**Figure 6:**
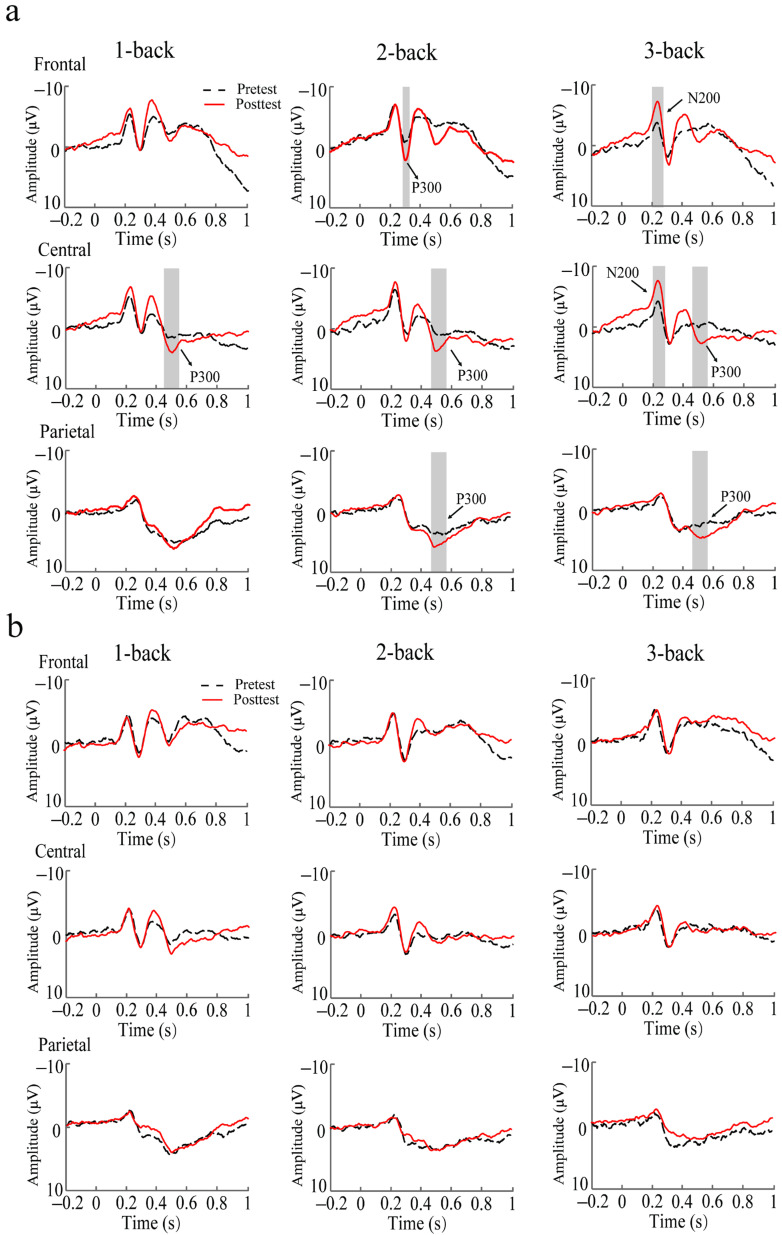
Event-related potentials (ERPs) pre- and post-test in the training (**a**) and control (**b**) groups. N200 and P300 amplitudes are shown in the frontal, central, and parietal regions. The gray rectangles highlight the marked differences between pre- and post-test results.

**Figure 7:**
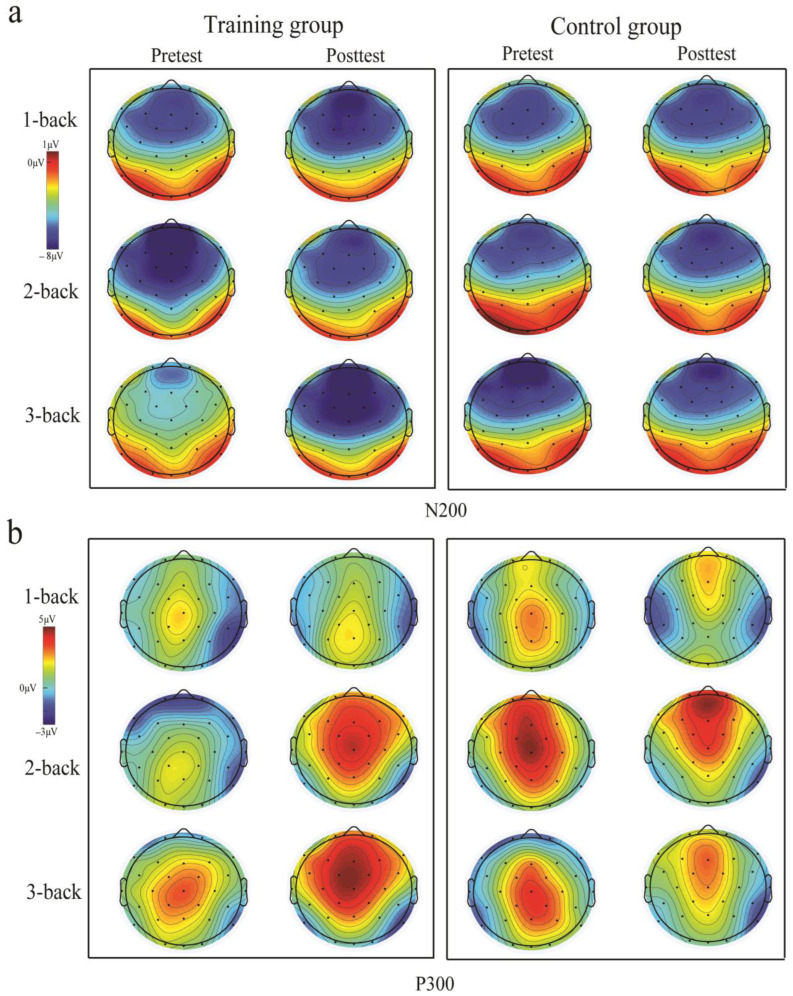
Topographic maps of N200 (**a**) and P300 (**b**) components under 1-, 2-, and 3-back conditions pre- and post-test in the training and control groups.

The N200 amplitude was assessed in the frontal (F3, F4, Fz), central (C3, Cz, C4), and parietal (P3, P4, Pz) regions. In the central region, there was a significant session × group × load interaction [*F*(2, 70) = 5.133, *p* < 0.008, η_p_^2^ = 0.135]. Post hoc analyses using pairwise comparisons with a Bonferroni correction indicated that there was no significant difference in the N200 amplitude pre-test between groups, *p* > 0.050. During the post-test, however, the training group presented significantly greater N200 amplitudes than the control group in the 1-back condition, *p* = 0.004. Notably, significant improvements post-test, compared to pre-test, in the 3-back condition were observed in the training group, *p* = 0.041, see Figure 5, Figure 6 and Figure 7. There was no change over time in the control group across all working memory loads, *ps* > 0.050. No other significant main effects or interactions were observed (*ps* > 0.050). In the central and parietal regions, no significant main effects or interactions were observed (*ps* > 0.050).

Then, the P300 amplitudes were assessed in these regions. In the frontal regions, a significant session × group interaction was observed [*F*(1, 35) = 4.228, *p* = 0.048, η_p_^2^ = 0.117]. Post hoc analyses using pairwise comparisons with a Bonferroni correction indicated a pronounced P300 amplitude enhancement post-test, compared to pre-test, in the training group, *p* = 0.053, see Figure 5, Figure 6 and Figure 7. However, a significant difference between post- and pre-test results in the control group was not observed, *p* > 0.050. In terms of sessions, the training group showed no significant difference in the P300 amplitude compared to the control group during the pre-test, *p* > 0.050; similarly, post-test, there no significantly greater P300 amplitude was evident in the training group compared to the control group, *p* > 0.050. No other significant main effects or interactions were observed (*ps* > 0.050). In the central regions, there was also a significant session × group interaction [*F*(1, 35) = 7.708, *p* = 0.009, η_p_^2^ = 0.189]. Post hoc analyses demonstrated that the training group showed significantly greater P300 amplitudes post-test, compared to pre-test, *p* = 0.008, see Figure 4, Figure 5 and Figure 6. There was no significant difference in these values between post- and pre-test results in the control group, *p* > 0.050. Moreover, post-test, the training group showed a significantly greater P300 amplitude than the control group, *p* = 0.013. Pre-test, a significant difference between the training and control groups was not observed, *p* > 0.050. No other significant main effects or interactions were observed (*ps* > 0.05). For the parietal region, no significant main effects or interactions were observed (*ps* > 0.05).

#### 3.2.2. Transfer Outcome Measures

**Figure 8:**
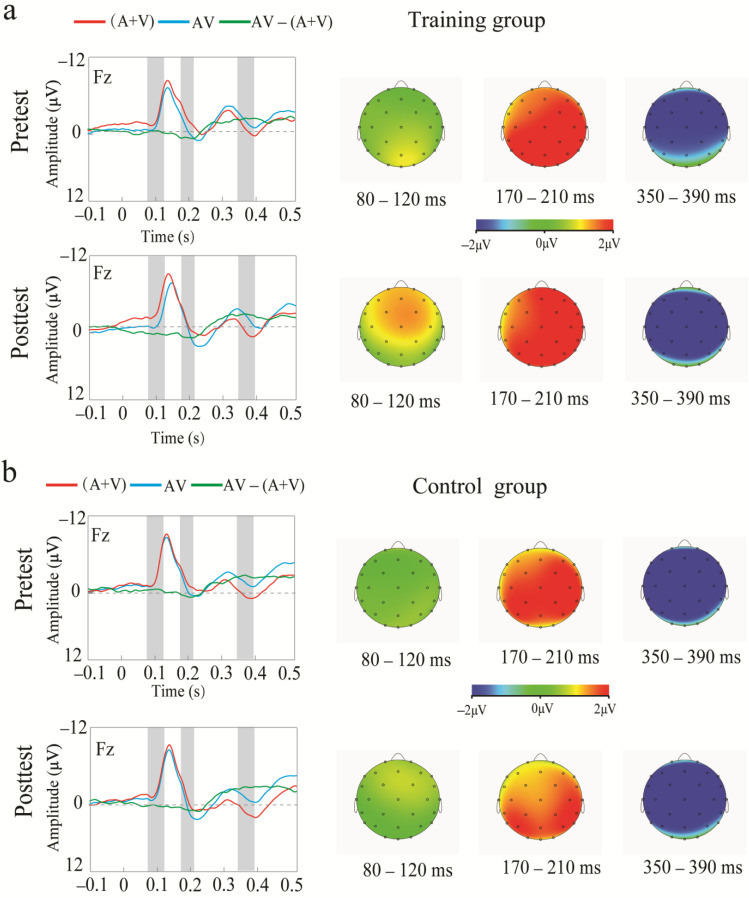
Grand-average event-related potentials of AV, A+V, AV − (A + V) and the topography map of audiovisual integrations in the training (**a**) and control (**b**) groups from 100 ms before stimulus onset to 500 ms after stimulus onset pre- and post-test.

At 80–120 ms, the ANOVA showed that the interaction between session and group was significant [*F*(1, 35) = 6.181, *p* = 0.018, η_p_^2^ = 0.147]. A post hoc analysis using pairwise comparisons with a Bonferroni correction showed that the post-test amplitude was greater than that the pre-test amplitude in the training group (*p* < 0.05), whereas such an improvement was not observed in the control group (*p* > 0.05). In terms of the session, the two groups showed no differences pre-test (*p* > 0.050); however, a higher amplitude was observed in the training group post-test (*p* = 0.026). Moreover, a significant three-way interaction was observed [*F*(2, 70) = 5.245, *p* = 0.007, η_p_^2^ = 0.127]. A post hoc analysis using pairwise comparisons with a Bonferroni correction suggested that there was a significant transfer effect in the frontal region. That is, the post-test amplitude was higher than that the pre-test amplitude in the training group, *p* = 0.037, see Figure 8. Such a transfer effect was not observed in the control group in the frontal region, *p* > 0.050. In the central region, the training group demonstrated a greater amplitude result post-test rather than pre-test (*p* = 0.006), whereas the control group did not present such a significant difference (*p* > 0.050). In the parietal region, significant differences between pre- and post-test results were not observed for either group, *ps >* 0.050. No other significant main effects or interactions were observed, *ps >* 0.050. At 170–210 ms, no significant main effects or interactions were observed, *ps >* 0.050. At 350–390 ms, the ANOVA showed a significant main effect of the region [*F*(2, 70) = 4.713, *p* = 0.012, η_p_^2^ = 0.116], indicating that the amplitudes in the frontal and central regions were significantly higher than that in the parietal region. However, no other significant main effects or interactions were observed, *ps >* 0.05.

## 4. Discussion

The current study investigated whether audiovisual n-back training induced a training effect on working memory performance and a transfer effect on audiovisual processing behavior. Regarding the training effect, the behavioral results showed that training led to an improved working memory performance with increased accuracy and decreased reaction time across 1-, 2-, and 3-back conditions. Moreover, the training group exhibited greater training gains in the 3-back condition compared to the 1- and 2-back conditions. The ERPs analysis showed that audiovisual n-back training led to the enhancement of the N200 amplitude in the 3-back condition over the frontal area and a higher P300 amplitude in the training group over the frontal and central areas. Regarding the transfer effect, the behavioral results showed that training did not induce significant differences in the accuracy and reaction time for the audiovisual processing task between pre- and post-test results. However, the ERP results showed that audiovisual integration in the frontal and central regions at 80–120 ms was enhanced in the training group, whereas such an effect was not observed in the control group.

### 4.1. Training Effect

#### 4.1.1. Behavioral Performance

The training effects appeared as greater accuracy and faster RT post-test across the 1-, 2-, and 3-back conditions in the training group. These results align with the previous studies that reported greater accuracy and faster RT following working memory training [6,37,38]. Moreover, we found that the training gain was significantly better in the 3- and 2-back conditions than in the 1-back condition. The available explanation is that a greater cognitive load may lead to greater audiovisual benefits [39]. Some researchers have supported this idea by examining whether audiovisual integration leads to a better working memory. Their results indicated improved accuracy on the most demanding tasks (2- and 3-back conditions) for audiovisual stimuli compared to visual or auditory stimuli, whereas such an improvement was not observed in the 1-back condition. This finding suggests that unimodal conditions may be sufficient for working memory processing when the load is low, but that the advantage of audiovisual benefits becomes obvious when the load increases and more resources are needed to complete the task [5]. Therefore, we inferred that better training effects in the 2- and 3-back conditions were related to increased audiovisual benefits under higher cognitive loads. Notably, we recruited college students who may possess better cognitive abilities than the general population [40]. In the 1-back condition, a ceiling effect may exist, resulting in no increases in training gains in this condition. 

#### 4.1.2. Neural Effects of Audiovisual n-Back Training

Our results show that audiovisual working memory training induces a higher amplitude for P300, consistent with the previous findings on working memory training in a single modality [17,20,41]. P300 is a well-established index that reflects working memory updates [20,23]. According to the context updating theory, the updating process monitors incoming information and replaces old information that is less relevant to the current task with new information that is more relevant to the current task, thus continuously revising the contents of working memory [42]. Increased P300 amplitudes may reflect a better updating ability engaged in working memory processing [20]. Neurocognitive models of the n-back task suggest that encoding, maintenance, and updating are necessary processes for working memory [43]. Encoding is critical to working memory processes because it significantly affects subsequent memory processes, such as maintenance and updating [21,44]. Some studies have reported faster working memory updating in an audiovisual working memory encoding condition compared with visual-only or auditory-only working memory encoding conditions [5]. This suggests that the presentation of audiovisual stimuli may elicit more effective encoding, improving the performance of later working memory processes. Therefore, we inferred that working memory training with audiovisual stimuli may also exert a positive effect on later updating processes, contributing to the enhancement of the P300 amplitude.

An enhanced N200 amplitude in the frontal region, especially in the most difficult 3-back condition, was also observed. This ERP component is associated with conflict monitoring and mismatch identification [19]. Mismatch identification was initially recognized in a sequential matching task, in which participants were required to judge whether a second stimulus matched or mismatched an initial stimulus [19]. The audiovisual n-back task in the current study involved a similar paradigm in which participants were asked to determine whether the current audiovisual stimulus matched or mismatched the previously audiovisual stimulus maintained in the working memory. The audiovisual n-back task in the current study involved a similar paradigm where participants needed to determine whether there was a mismatch between a currently previously presented stimulus maintained in the working memory. According to memory trace theory, the presentation of audiovisual stimuli leads to a strong audiovisual memory trace during encoding, and the maintenance of the working memory is facilitated due to the factor that it also activates audiovisual memory representation [45]. Because mismatch identification requires a comparison between the currently presented stimulus and the stimulus maintained in the working memory, the facilitation of working memory maintenance may increase mismatch identification, reflected by the enhancement of the N200 amplitude. In addition, conflict monitoring has also been involved in working memory processing, especially with lure trials [46]. The lure trial in n-back conditions is the same as a previously presented stimulus, but is not the matched trial (e.g., 3-back, the second animal (cat) in the stimuli stream “dog–cat–cow–cat” is the lure trial), which may lead to stimulus familiarity and induce a strong conflict effect [18]. Their results confirm that greater N200 amplitudes exist in the training group post-test, indicating that conflict monitoring processes are likely engaged in the n-back task with lure trials [18]. However, the task used in our current training solely involved audiovisual facilitations and did not include lure trials. Therefore, the enhanced N200 amplitude may not be related to improved conflict monitoring.

Notably, the current study found that only the 3-back condition was affected by training, as the N200 amplitude was significantly increased in the training group compared to the control group, consistent with the previous studies showing that training induced significant neural alterations in the 3-back condition [41]. A possible explanation is that the general executive control process is engaged in more demanding tasks [47]. Early studies compared neural activation across different visual n-back conditions (0-, 1-, 2-, and 3-back) and found that more brain areas were activated as the difficulty was increased (i.e., increasing n). Moreover, the dorsolateral prefrontal cortex (DLPFC) only became involved in the task only more demanding conditions (e.g., 2- and 3-back) [48]. Because the activation of the DLPFC is associated with executive functions, this finding indicates that executive functions are specifically engaged under more demanding conditions [49]. We inferred that our training may have improved executive functions, as reflected by the greater N200 amplitude in the 3-back condition after training.

Overall, audiovisual n-back training can induce the enhancement of N2 and P3. Previous research has suggested that the component of P3 is related to updating and N2 is related to mismatch identification in the N-back task [19,20]. The enhancement of these two neural components after training offered neural evidence that working memory performance was improved.

### 4.2. Transfer Effect

With respect to the transfer effect, our study showed that audiovisual working memory training induced enhanced audiovisual integration (at 80–120 ms) in the frontal and central regions. Audiovisual integration is interplayed by both early bottom-up and late-top-down processing [50]. The amount of available cognitive resources for audiovisual integration may determine the weights of bottom-up and top-down processing [51]. Some evidence is derived from the research that investigated how memory load modulates neural oscillations during audiovisual integration. These results showed that audiovisual integration under a high memory load with scarce cognitive resources requires greater top-down processing, reflected by the engagement of theta and alpha oscillations [51]. In our research, the transfer task was a simple discrimination task that may have required fewer cognitive resources, suggesting that audiovisual integration was largely governed by bottom-up processing. Moreover, some researchers have explored the latency of bottom-up processing. It appears that the early latency, around 100 ms (e.g., N1), reflects a relatively bottom-up process [50,52]. Therefore, we infer that the enhancement of audiovisual integration at 80–120 ms in our research reflects an improvement in bottom-up processing.

Audiovisual n-back training facilitates audiovisual processing, reflected by enhanced audiovisual integration at an early stage after training. This finding supports the idea that a transfer primarily occurs when there is an overlap between the brain regions involved in training and transfer tasks [28]. The working memory and audiovisual processing were linked to the left intraparietal region. In addition, some researchers found that there was a positive relationship between working memory and audiovisual processing [32]. That is, greater working memory capacities may inhibit the decline in the audiovisual process. Therefore, audiovisual n-back training improved the working memory, consequently enhancing the ability of audiovisual processing.

### 4.3. Limitations

Several limitations of the current research should be addressed. First of all, given the fact that the behavioral results for the transfer effect on audiovisual processing were negative, this effect was only discussed at the neural level. The relationship between neural and behavioral effects was not clarified. Therefore, in the following study, we should determine why there was a dissociation between behavioral and neural results. Second, the possibility that motivation and familiarity with the training procedure may influence the performance cannot be ruled out. Third, we only compared the training and control groups regarding younger adults. Training may potentially lead to the development of effective interventions to improve cognitive and daily-life functions for older adults or individuals with cognitive impairments. Future studies should focus on these populations as they suffer from the age-related issue of the deterioration of working memory.

## 5. Conclusions

The current study aimed to investigate the training and transfer effects of audiovisual working memory tasks, including both behavior and neural outcomes. The results demonstrate that training leads to an improved working memory performance, as well as the enhancement of N200 and P300 components. Moreover, the training not only successfully improved working memory, but also affected audiovisual processing, reflected by the enhanced audiovisual integration at earlier stage. This implied that higher cognitive functions influenced lower cognitive functions. In summary, these results can provide insights into the neural mechanisms underlying audiovisual working memory training and lead to a more comprehensive understanding of working memory design. Audiovisual working memory training may be a reliable training method, providing new ideas for the design of cognitive training and clinical treatment practices.

## Figures and Tables

**Table 1:** Performance of the transfer task. Mean reaction times and accuracy results for training and control groups pre- and post-test for audiovisual discrimination tasks. Standard deviations are presented in parentheses.

	Auditory		Visual		Audiovisual	
	Pre-Test	Post-Test	Pre-Test	Post-Test	Pre-Test	Post-Test
Training group						
Accuracy (%)	76 (20)	87 (13)	76 (19)	83 (14)	94 (6)	95 (6)
RT (ms)	420 (34)	413 (28)	416 (27)	415 (25)	382 (37)	373 (29)
Control group						
Accuracy (%)	74 (25)	77 (17)	72 (20)	73 (18)	92 (9)	95 (4)
RT (ms)	424 (38)	424 (33)	422 (31)	423 (24)	394 (43)	386 (39)

## Data Availability

The data related to the study can be obtained from the corresponding author on reasonable request.
